# TSDGNet: Multiscale Temporal and Dual-Graph Learning for Pathological Gait Classification Using Wearable Inertial Sensors

**DOI:** 10.3390/bios16100584

**Published:** 2026-10-18

**Authors:** Shi Ye, Zihao Xia, Boyi Li, Donghao Wu, Chi Liu, Liyong Ma, Shuzheng Shi

**Affiliations:** 1 School of Mechanical Engineering, Hebei University of Architecture, Zhangjiakou 075000, China; ys2025225122@hebiace.edu.cn (S.Y.); 2024225118@hebiace.edu.cn (Z.X.); lby2025225105@hebiace.edu.cn (B.L.); wzx2124@hebiace.edu.cn (D.W.); ssz1314@hebiace.edu.cn (S.S.); 2 School of Mechanical and Electrical Engineering, Changsha University, Changsha 410199, China; liuchi@ccsu.edu.cn; 3 School of Mechanical Engineering and Automation, Beihang University, Beijing 100191, China; 4 Hebei Technology Innovation Center for Intelligent Production Line of Prefabricated Building Components, Zhangjiakou 075000, China

**Keywords:** wearable inertial sensors, pathological gait recognition, multisensor fusion, lower limb disease, anatomical priors

## Abstract

Wearable inertial sensors offer a portable means of assessing gait abnormalities associated with neurological and orthopedic disorders. However, intersubject variability, overlapping gait patterns, and class imbalance complicate multiclass recognition. This study presents the Temporal Structural Dual Graph Network (TSDGNet) for pathological gait classification using inertial measurement unit (IMU) signals. Four IMUs positioned on the head, lower back, and both feet provide acceleration and angular velocity signals during walking and turning. The framework combines multiscale temporal feature extraction with graph-based modeling of sequence-level relationships among sensor representations. Two fixed prior graphs encode anatomical and symbiotic relationships, while trainable graph attention and context-guided gated residual refinement adapt sensor-node representations. Class-balanced optimization is incorporated to reduce training bias toward majority classes. Subject-independent evaluation was performed on a public clinical dataset comprising healthy controls and seven neurological or orthopedic disease groups. Comparisons with representative baseline and advanced models showed competitive classification performance. Ablation analyses examined the contributions of temporal modeling, structural relationship learning, and class-balanced optimization. These findings support the potential of combining wearable inertial sensing with temporal and graph-based learning for objective classification of pathological gait.

## 1. Introduction

Human gait is a noninvasive physiological marker that objectively reflects neuromuscular coordination and musculoskeletal function [1]. Therefore, quantitative gait analysis has become increasingly important for the early screening, clinical diagnosis, and long-term monitoring of lower limb disorders [2]. Although laboratory motion capture systems can provide highly accurate biomechanical measurements, their high cost, limited portability, and dependence on dedicated environments greatly restrict their routine clinical use and large-scale application [3]. In contrast, wearable inertial measurement units (IMUs) provide a portable, economical, and easily deployable alternative, making them suitable for continuous gait monitoring in both clinical and real-world settings [4].

A major advantage of IMU-based gait analysis is its ability to capture natural walking behavior in daily life, including straight walking, turning, and task transitions, rather than being limited to controlled laboratory conditions [5]. This is especially valuable for telemedicine, rehabilitation assessment, and long-term disease management, where frequent and unobtrusive monitoring is often needed [6]. Meanwhile, advances in wearable sensing have promoted the standardization of data acquisition, signal segmentation, feature extraction, and model evaluation, providing a solid basis for clinical gait recognition [7].

In recent years, deep learning has been widely introduced into IMU-based gait analysis to reduce dependence on handcrafted features and improve the modeling of complex temporal dynamics [8]. Deep neural networks can learn informative representations directly from raw or minimally preprocessed sensor signals [9] and have shown promising performance in gait event detection, disease screening, and functional impairment recognition [10]. However, most existing studies still focus on relatively simple settings, such as binary classification between healthy individuals and patients or recognition of a single disease [11]. In real clinical practice, gait analysis often requires joint recognition across multiple lower limb disorders within highly heterogeneous patient populations [12].

This task is particularly challenging because lower limb disorders often present substantial heterogeneity, including overlapping symptoms, compensatory movement strategies, and individualized gait adaptations [13]. In addition, clinically collected datasets often suffer from inter-subject variability, severe class imbalance, and inconsistencies in acquisition protocols, all of which can weaken model robustness and generalizability [14]. For clinically meaningful evaluation, validation across independent subjects is essential, since any leakage of subject data between training and testing can lead to overestimated performance [15]. At the same time, long-tail class distributions tend to bias models toward majority classes, reducing sensitivity to minority pathological patterns even though these rare categories may carry important clinical value [16].

From a modeling perspective, many existing IMU-based methods treat multi-sensor signals as ordinary multivariate time series and mainly rely on temporal encoders for representation learning [17]. Although effective in modeling temporal dynamics, these architectures often fail to fully exploit the anatomical relationships inherent in multi-node sensor layouts. Graph learning has shown strong potential in this regard because it can incorporate structural prior knowledge and explicitly characterize dependencies among body segments [18]. However, directly applying generic graph modeling techniques to clinical IMU signals remains difficult, since IMU data provide only sparse representations of limb motion and pathological gait changes are often subtle, entangled, and highly variable across individuals [19].

To address these challenges, this study focuses on multiple disease recognition of lower limb disorders using wearable IMU data and develops a computational framework that integrates pathology-aware temporal modeling, dual-branch graph learning, and disease-aware optimization. The proposed framework is designed to capture both fundamental gait rhythms and disease-related dynamic variations, while refining node features through fixed anatomical and symbiotic prior graphs with attention-based propagation and context-guided gated residual fusion. In this way, it aims to alleviate the adverse effects of long-tail distributions and pathological similarity, thereby providing a structured computational approach for multiclass pathological gait classification from wearable IMU signals.

The main contributions of this study are summarized as follows:

(1) Multiscale temporal modeling and context construction. A temporal backbone combines multiple convolutional kernel sizes with adaptive branch weighting to extract gait features at different temporal scales. Global temporal average pooling and a trainable linear projection construct a pathological context vector for conditioning subsequent graph refinement.
(2) Dual-branch graph learning with fixed priors and context-guided residual refinement. Two predefined graphs provide complementary anatomical and coordination priors for cross-sensor feature aggregation. Topology-constrained attention refines node representations, and a context-guided gate combines the resulting residual feature updates.
(3) Training optimization for disease confusion: A targeted learning mechanism is introduced to mitigate the problems of multi-class data imbalance and frequent misclassification among similar diseases. For disease categories with relatively low prevalence but high clinical importance, this mechanism improves the model’s recognition performance.

## 2. Related Work

Wearable IMUs have received increasing attention in gait analysis because of their low cost, ease of deployment, and ability to support continuous monitoring. With the accumulation of clinical gait datasets and the development of intelligent analysis methods, IMU-based gait recognition has gradually evolved from handcrafted feature design to automatic representation learning from raw signals. Existing studies mainly concern four aspects: clinical IMU gait assessment, deep learning for pathological gait recognition, temporal and graph modeling for gait analysis, and methodological limitations under clinical constraints.

### 2.1. Clinical IMU Gait Assessment

In clinical gait research, the main value of IMUs lies in their ability to provide quantifiable and repeatable descriptions of disease-related movement abnormalities. Previous studies have contributed both to the construction of clinical gait datasets and to the refinement of analysis pipelines. Voisard et al. [20] established a clinical gait database covering healthy individuals as well as patients with neurological and orthopedic disorders, providing a systematic foundation for cross-disease gait analysis. At the methodological level, the open-source gaitmap tool proposed by Kuderle et al. [21] integrated sensor calibration, gait event detection, gait segmentation, and feature processing, thereby improving reproducibility and comparability across studies.

As IMU-based research has moved from laboratory settings to practical clinical scenarios, its application scope has further expanded. Ullrich et al. [22] predicted fall risk in patients with Parkinson’s disease using inertial gait data collected in daily life, showing that natural gait behavior contains valuable clinical information. Meng et al. [23] found that, compared with steady straight walking, turning-related inertial gait features are more sensitive to early Parkinson’s disease, suggesting that pathological information may be concentrated in specific task segments rather than uniformly distributed across the gait cycle. For more refined assessment, Liu et al. [24] developed a low-cost IMU system for Parkinson’s disease subtype classification and stage evaluation, showing that even a limited sensor configuration can support detailed clinical discrimination.

Beyond Parkinson’s disease, IMUs have also shown potential in other pathological gait studies. Dammeyer et al. [25] demonstrated that inertial gait patterns can distinguish different orthopedic disorders, indicating that gait signals are also informative for musculoskeletal diseases. Liuzzi et al. [26] combined inertial sensors with machine learning to estimate dynamic balance and gait adaptability in neurological disorders, suggesting that gait analysis can support both disease recognition and functional status quantification. Lin et al. [27] used instrumented insoles and deep learning to predict multiple balance scale scores from gait data collected in natural environments, indicating that long-term gait recordings can complement conventional clinical scales. In addition, Liu et al. [28] proposed a fuzzy logic method for lower limb IMU gait event detection, providing more stable support for subsequent feature extraction and disease recognition.

Overall, previous studies have clearly demonstrated the feasibility and practical value of IMUs in clinical gait assessment. However, most existing work still focuses on a single disease or a single task, and there remains insufficient exploration of how to jointly model subtle differences across multiple lower limb disorders within a unified framework.

### 2.2. Deep Learning for Pathological Gait Recognition

Traditional pathological gait recognition methods usually rely on manually extracted time domain, frequency domain, or statistical features, followed by classifiers such as support vector machines, random forests, or gradient boosting models. Although these approaches can be useful under small sample conditions, they depend heavily on feature engineering and often have limited ability to capture the nonlinear dynamics of complex pathological gait. To overcome these limitations, recent studies have increasingly adopted deep learning to learn discriminative representations directly from raw or minimally preprocessed sensor signals.

Representative studies have shown the advantages of this direction. Turner and Hayes [29] used wearable sensors and deep learning to classify subtle gait abnormalities, demonstrating the ability of deep models to distinguish fine-grained gait changes. Hwang et al. [30] further considered joint injury-related factors in the recognition process, indicating that pathological gait analysis should account not only for surface-level signal differences but also for underlying biomechanical impairment mechanisms. Thakur and Biswas [31] introduced an attention mechanism into hemiplegic gait prediction using smartphone sensor data, showing that attention-based enhancement can improve pathological gait representation learning.

Despite these advances, most existing studies still focus on binary classification between healthy individuals and patients, or on recognition and grading for a single disease. In contrast, real clinical multiple disease recognition involves more complex disease overlap, symptom mixing, and inter-subject variability, which makes it difficult for conventional deep networks alone to fully distinguish subtle pathological differences.

### 2.3. Temporal and Graph Modeling for Gait Analysis

Gait is a dynamic behavior with both temporal and structural properties. Its discriminative information is reflected not only in temporal evolution, such as rhythm and phase transitions, but also in coordination relationships among different body segments. Therefore, treating multi-sensor gait signals only as ordinary multivariate time series is often insufficient for characterizing coupling patterns across body segments in pathological gait.

To address this issue, researchers have introduced structural modeling into gait recognition. Yan et al. [32] proposed a spatiotemporal graph convolutional network with spatial and temporal attention, which maps multi IMU gait data into a skeleton-like graph structure and models dependencies among sensor nodes on the graph topology, thereby capturing coordinated movement patterns among body parts more effectively. This suggests that graph modeling is not merely a supplement to temporal modeling, but a representation that is more consistent with the organization of human movement. For pathological gait, abnormalities are often expressed not only as isolated local changes but also as reorganized coordination across joints and body segments, which makes structural relationship modeling especially meaningful.

However, effectively combining temporal modeling and structural modeling for clinical multiple disease gait recognition remains difficult. Clinical gait signals are often highly nonstationary because of rhythm changes, compensatory behaviors, and task switching. At the same time, relationships among body segments are constrained by both anatomical structure and deviations induced by disease. How to preserve anatomical priors while learning disease-driven spatiotemporal interaction patterns therefore remains an open challenge.

### 2.4. Limitations of Existing Methods

In addition to model architecture, the characteristics of clinical data also impose important constraints on pathological gait recognition. To address limited training data, Zhang et al. [33] combined few-shot learning with generative learning for real-time pathological gait prediction on wearable devices, highlighting the importance of model generalization under data-scarce conditions. However, such methods mainly focus on insufficient sample size and do not specifically address the complex confusion among multiple diseases.

Another important issue is interpretability. Xiang et al. [34] emphasized that explainable artificial intelligence for gait analysis should not only determine whether a model is correct, but also reveal which time segments, gait phases, or relationships among body segments contribute most to the final decision. This is essential not only for model acceptance in clinical practice but also for supporting disease understanding and rehabilitation planning. In addition, class imbalance remains a common problem in clinical gait data. Xu and Li [35] showed that under long-tail distributions, models can easily become biased toward majority classes if the training objective is not properly constrained, thereby weakening recognition of minority disease categories.

In summary, although existing studies have made substantial progress in clinical gait data construction, deep feature learning, spatiotemporal modeling, and training strategy design, several important limitations remain in multiple-class lower limb disease classification. Pathological temporal dynamics are often modeled too coarsely, structural relationships among body segments are still underused, and practical issues such as class imbalance, cross-subject distribution shift, and clinical interpretability are rarely handled in a unified manner. Therefore, developing a unified framework that can jointly address temporal dynamics, structural relationships, class imbalance, and clinical interpretability remains a key direction for multiple disease gait recognition of lower limb disorders.

## 3. Method

### 3.1. Overview of the Proposed Method

This study proposes a unified framework that integrates multi-scale temporal modeling, dual-branch graph learning, and class-balanced optimization for multi-class lower-limb disease gait recognition. The framework first extracts temporal features from raw inertial signals, then employs a dual-branch graph learning module to model the relationships among different body segments, and finally performs disease classification through feature fusion and class-balanced optimization.

**Figure 1:**
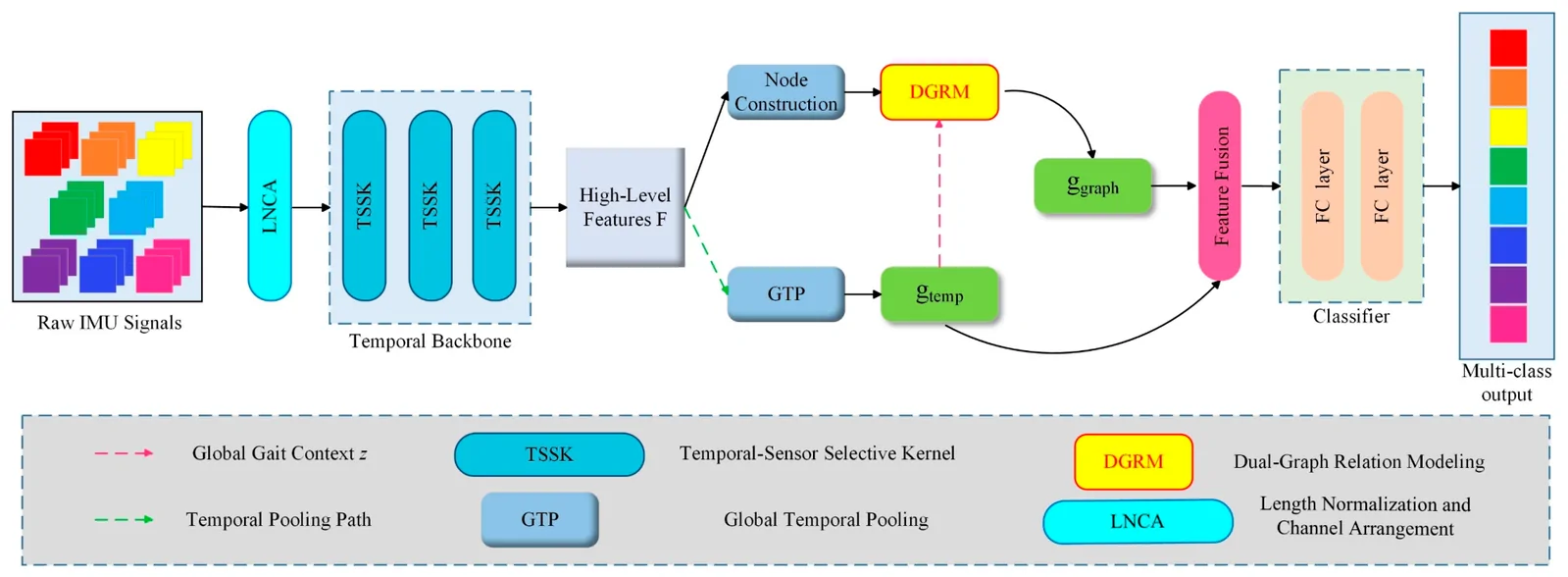
Overall architecture of the proposed TSDGNet for multi-class lower-limb disease gait recognition.

Specifically, the model first employs a multi-scale temporal backbone to encode the raw inertial signals and extract high-level temporal representations that can simultaneously capture local abnormal variations and global temporal patterns. Subsequently, according to the deployment positions of the sensors on the body, the temporal features are mapped into node representations, and dual-branch graph learning is conducted on both the anatomical prior graph and the symbiotic relation graph to further characterize the association patterns among different body segments. Finally, the temporal representation and graph-level structural representation are fused, and the model is trained with class weighting, weighted sampling, and class-balanced optimization strategies, thereby improving its ability to distinguish imbalanced categories and easily confused disease classes. The overall architecture of the proposed method is illustrated in Figure 1.

### 3.2. Input Representation and Temporal Feature Extraction

Let the raw input tensor of a single sample be denoted by *X* ∈ *ℝ^T^*^×*M*×*D*^, where *T* represents the original temporal length, *M* denotes the number of sensor nodes, and *D* denotes the number of inertial signal channels associated with each node. In the task considered in this paper, the number of sensor nodes is 4, and each node contains 6 inertial signal channels. Therefore, the raw sample can be further written as *X* ∈ *ℝ^T^*^×4×6^. This means that, at each sampling instant, the system synchronously records six-dimensional motion information from four body locations.

Because the effective lengths of different samples are not identical, directly feeding them into the network would not only make mini-batch training unstable, but could also allow sequence length differences themselves to interfere with feature learning. To address this issue, all samples are first normalized to a fixed length *S*. Let the preprocessed sample be denoted by $\tilde{X}$. Its elementwise form is defined as:

(1) $${\tilde{X}}_{t,m,d}=\left\{\begin{array}{ll} X_{t+s,m,d}, & L\geq S,1\leq t\leq S\\ X_{t,m,d}, & L<S,1\leq t\leq L\\ 0, & L<S,L<t\leq S \end{array}\right.$$

where ${\tilde{X}}_{t,m,d}$ denotes the value of the preprocessed sample at time step *t*, sensor node *m*, and channel *d*; *X_t_*_,*m*,*d*_ denotes the value at the corresponding position in the original sample; *L* is the effective length of the current sample; *S* is the target sequence length; and *s* is the starting position of center cropping. When the original sample length is no shorter than the target sequence length, a continuous segment of length *S* is cropped from the center. When the original sample length is shorter than *S*, the valid original segment is retained and zero-padded at the end until the sequence reaches length *S*.

After length normalization, the node dimension and channel dimension are flattened to form the input tensor *x* for the temporal backbone network. The total number of channels is jointly determined by the number of sensor nodes and the number of channels per node. In this study, since there are 4 sensor nodes and each node contains 6 channels, the total number of channels after flattening is 24. That is, the input tensor *x* has the shape *ℝ*^24×^*^S^*. This representation offers two advantages. On the one hand, it preserves synchronized information from multiple sensor nodes within the retained temporal segment. On the other hand, it enables one-dimensional convolution to directly extract cross-temporal dynamic features within a unified channel space.

**Figure 2:**
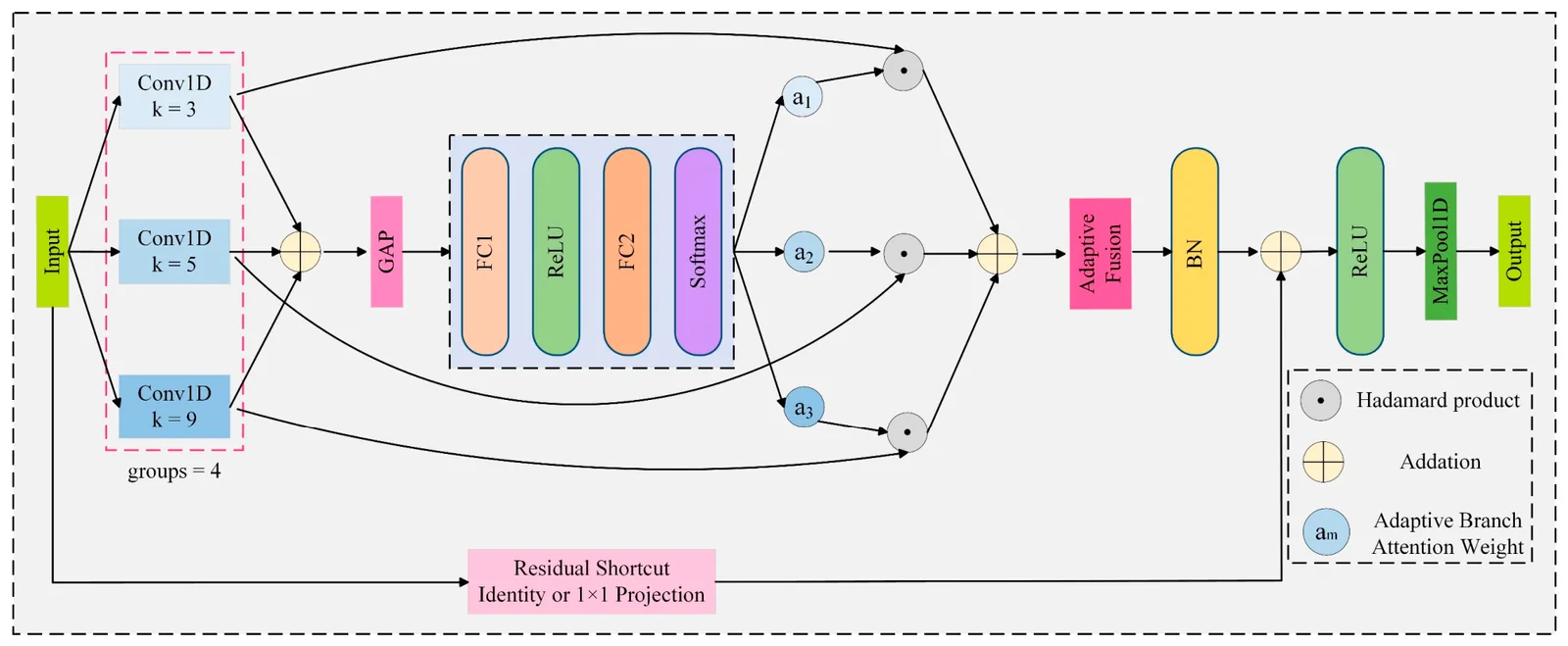
Internal structure of the TSSKBlock, including multi-branch temporal convolutions, adaptive branch weighting, residual shortcut, and feature fusion.

For temporal modeling, we adopt a backbone network composed of three TSSKBlocks. The three layers produce output channels of 64, 128, and 256, respectively. Each layer employs multi-branch convolutions with kernel sizes of 3, 5, and 9, together with a grouped convolution strategy organized by sensor node. The motivation is that gait abnormalities associated with different diseases are often distributed across different temporal scales. Short-term local fluctuations, medium-length dynamic segments, and rhythmic variations over longer temporal ranges may all contain important discriminative cues. If only a single-scale convolution is used, the model’s ability to adapt to multiclass pathological gait patterns is likely to be limited. The internal structure of the TSSKBlock is shown in Figure 2.

Let *U* denote the input feature of a certain layer, and let the convolution mapping of branch *m* be denoted by Φ*_m_*. Then the output of that branch can be written as

*Y_m_* = Φ*_m_*(*U*) (2)

where *Y_m_* denotes the output feature of the scale branch *m*, and Φ*_m_* denotes a one-dimensional convolution mapping with kernel size *k_m_*. In this study, *k_m_* ∈ {3,5,9}, corresponding to short, intermediate, and long temporal receptive fields, respectively.

To avoid forcing all samples to rely on a fixed temporal scale, we introduce an adaptive fusion mechanism across different convolution branches. The fused output is defined as:

(3) $$Y=\sum_{m=1}^{3}a_{m}⊙Y_{m},\;\sum_{m=1}^{3}a_{m}=1$$

where *Y* denotes the final output of the current layer, *a_m_* denotes the adaptive weight assigned to branch *m*, and ⊙ denotes element-wise multiplication for channel reweighting. These weights are automatically generated from the global statistical information of the input features. As a result, the network can adaptively determine which temporal scale should be emphasized for a particular gait pattern. Through this multi-scale and adaptive temporal encoding strategy, the model can obtain more stable dynamic representations when dealing with different categories, different individuals, and different levels of pathology.

After the three-layer temporal backbone, a high-level feature representation *F* is obtained. Let *f_τ_* denote the high-level feature vector at temporal position *τ*, where *τ* ranges from 1 to the temporal length *T*^′^ after downsampling. Then the global temporal representation and the global gait context vector are defined as:

(4) $$g_{\mathrm{temp}}=\frac{1}{T^{\prime }}\sum_{\tau =1}^{T^{\prime }}f_{\tau },\;z=W_{p}g_{\mathrm{temp}}$$

where *g_temp_* denotes the global temporal representation, *z* denotes the global gait context vector, and *W_p_* denotes a trainable linear projection matrix. The context vector provides a sequence-level summary of the encoded temporal features and conditions subsequent graph refinement.

### 3.3. Dual-Branch Graph Learning with Fixed Priors and Context-Guided Residual Refinement

Although the temporal backbone can effectively extract dynamic patterns, relying solely on temporal dynamic features remains insufficient for gait recognition with multi-location inertial sensors. This is because different sensors correspond to different body parts, and these body parts are naturally linked through anatomical connections and motion coordination relationships. Gait abnormalities are not merely reflected as changes in the signal morphology of a single node, but are more often manifested as changes in the coordination patterns among different body segments. Therefore, to distinguish multiple lower-limb diseases more accurately, conventional multivariate time-series modeling alone is usually inadequate, and the relationships among different body parts should also be incorporated into the model.

Motivated by this observation, a dual-graph structural modeling module is constructed on top of the high-level temporal features. Specifically, the output features from the last layer of the backbone are first reorganized according to the four sensor nodes and then averaged along the temporal dimension to obtain node-level representations. Let *f_i_*_,*τ*_ denote the feature vector of node *i* at temporal position *τ*. The graph-space representation of this node is defined as:

(5) $$h_{i}=W_{n}\left(\frac{1}{T^{\prime }}\sum_{\tau =1}^{T^{\prime }}f_{i,\tau }\right)$$

where *h_i_* denotes the graph-space representation of node *i*, and *W_n_* is the node projection matrix. The node feature matrix formed by all node vectors is denoted by *H*. In this study, the graph contains four nodes corresponding to the head, lower back, left foot, and right foot, respectively. For each input sequence, Equation (5) produces one temporally aggregated feature vector per sensor node. The graph module then models relationships among these sequence-level sensor representations.

To simultaneously represent stable anatomical relationships and disease-induced abnormal coupling patterns, two prior graphs with different semantics are explicitly defined. The first one is the anatomical prior graph:

(6) $$A_{\mathrm{body}}=\left[\begin{array}{cccc} 1 & 1 & 0 & 0\\ 1 & 1 & 1 & 1\\ 0 & 1 & 1 & 0\\ 0 & 1 & 0 & 1 \end{array}\right]$$

where *A_body_* denotes the anatomical prior adjacency matrix. This matrix emphasizes the lower back as the central node, with major connections to the head and both feet, thereby reflecting the relatively stable topological organization of the human body.

**Figure 3:**
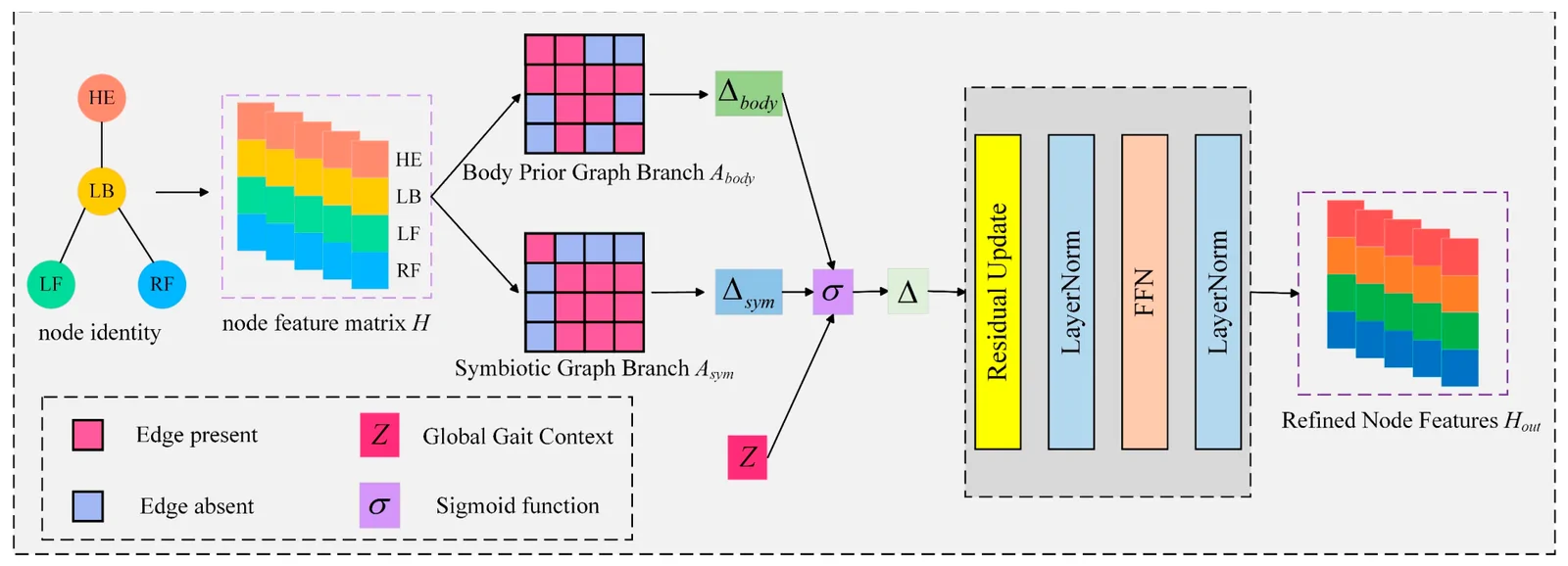
Pathology context-guided dual-graph refinement module. Node features are refined through the anatomical prior graph and the symbiotic graph, followed by gated residual fusion and feed-forward updating.

The second graph is the symbiotic relationship graph:

(7) $$A_{\mathrm{sym}}=\left[\begin{array}{cccc} 1 & 0 & 0 & 0\\ 0 & 1 & 1 & 1\\ 0 & 1 & 1 & 1\\ 0 & 1 & 1 & 1 \end{array}\right]$$

where *A_sym_* denotes the symbiotic adjacency matrix. Unlike the anatomical graph, this graph emphasizes the coordinated relationships between the lower back and the two lower limbs, as well as between the left and right lower limbs, thereby providing a structural space for representing compensatory behaviors and abnormal coordination patterns that commonly arise under pathological conditions. The pathology context-guided dual-graph refinement process is illustrated in Figure 3. Both adjacency matrices are predefined and remain fixed throughout training. The trainable components comprise the attention projections, the context-guided gating mappings, and the residual scaling coefficient.

During graph propagation, topology-constrained multi-head self-attention is applied separately on the two graphs, yielding two sets of updated node representations:

*H_body_* = 𝒜(*H*,*A_body_*), *H_sym_* = 𝒜(*H*,*A_sym_*) (8)

where 𝒜(⋅) denotes the adjacency-constrained multi-head self-attention mapping, *H_body_* is the updated node representation obtained on the anatomical graph, and *H_sym_* is the updated node representation obtained on the symbiotic graph. Because the attention propagation is strictly constrained by the adjacency structure, node information cannot spread freely along unreasonable paths. As a result, this mechanism preserves stronger structural consistency than fully connected attention.

To explicitly characterize the changes introduced by the two graph propagation processes relative to the original node states, two residual increments are further defined as:

Δ*_body_* = *H_body_* − *H*, Δ*_sym_* = *H_sym_* − *H* (9)

where Δ*_body_* denotes the node increment under the anatomical graph view, and Δ*_sym_* denotes the node increment under the symbiotic graph view. These residual terms represent node-feature updates produced by graph propagation, rather than newly constructed edges or changes to the adjacency matrices.

After obtaining the two residual terms, the global gait context vector is introduced to modulate their averaged structural increment through a sample-specific gate. Let *Z* denote the matrix obtained by expanding the global gait context vector *z* to all nodes. The gate weights are defined as:

(10) $$M=\sigma \left(W_{2}\tanh\left(W_{1}\left[\begin{array}{c} \Delta_{\mathrm{body}}\\ \Delta_{\mathrm{sym}}\\ Z \end{array}\right]\right)\right)$$

where *M* denotes the gate weights, *σ*(⋅) is the Sigmoid function, where *W*_1_ and *W*_2_ are the linear mapping parameters in the gating network. The gate weights are determined jointly by the graph residual features and the sequence-level context derived from the temporal backbone.

Accordingly, the gated integrated structural increment can be written as:

(11) $$\Delta =\frac{1}{2}M⊙(\Delta_{\mathrm{body}}+\Delta_{\mathrm{sym}})$$

where Δ denotes the integrated structural increment. Furthermore, a learnable scaling coefficient is introduced to control the influence strength of the graph residual on the node representation.

Combined with layer normalization and a feed-forward mapping, the final node representation is obtained as:

(12) $$\hat{H}=\mathrm{LN}(H+\alpha \Delta ),\;H_{\mathrm{out}}=\mathrm{LN}\left(\hat{H}+F(\hat{H})\right)$$

where $\hat{H}$ denotes the intermediate node representation after residual updating, *H_out_* denotes the refined node representation, *α* is the learnable residual scaling coefficient, *LN*(⋅) denotes layer normalization, and ℱ(⋅) denotes the feed-forward mapping.

After graph refinement, all nodes are averaged to obtain the graph-level representation *g_graph_*. This representation is then fused with the global temporal representation *g_temp_* to form the final joint representation and classification output:

(13) $$g_{\mathrm{graph}}=\frac{1}{N}\sum_{i=1}^{N}h_{i}^{\mathrm{out}},\;u=\left[\begin{array}{c} g_{\mathrm{temp}}\\ g_{\mathrm{graph}} \end{array}\right],\;o=\Psi (u)$$

where *g_graph_* denotes the graph-level representation, $h_{i}^{\mathrm{out}}$ is the refined representation of node *i*, *u* denotes the final joint representation, *o* denotes the class prediction output of the classifier, and Ψ(⋅) denotes a classifier composed of two fully connected mappings. The global temporal representation summarizes features extracted by the temporal backbone, while the graph-level representation aggregates cross-sensor relationships among the time-averaged node features. Their fusion combines these complementary representations for multiclass pathological gait classification.

### 3.4. Training Optimization for Disease Confusion

Beyond the model architecture itself, the design of the training objective also has a direct impact on multi-disease recognition performance. Clinical data generally exhibit class imbalance. If the model is trained directly with a conventional empirical risk minimization strategy, it tends to become biased toward classes with more samples, thereby reducing its sensitivity to minority disease categories. To address this issue, the proposed method does not rely on manual augmentation tailored to any specific disease during training. Instead, class weights are automatically generated according to the class frequencies in the training set. Let the number of samples in class *c* be *n_c_*. The corresponding class weight is defined as:

(14) $$w_{c}=\frac{1-\beta }{1-\beta^{n_{c}}+\varepsilon }$$

where *w_c_* denotes the weight of class *c*, *β* is the balancing factor, and *ε* is a small constant introduced to prevent numerical instability.

At the sampling stage, a weighted random sampling strategy is further employed so that minority class samples have a higher probability of being selected in each training epoch. This design is important because introducing class weights only in the loss function can increase the penalty assigned to errors on minority classes, but it cannot fundamentally resolve the problem that such samples appear too infrequently in mini-batch training. In contrast, weighted sampling improves the visibility of these samples from the perspective of data flow, allowing the model to encounter minority and hard samples more frequently during parameter updates.

For the loss function, a class-weighted focal loss is adopted. The weighted cross-entropy is first defined as:

(15) $$L_{\mathrm{ce}}=-\sum_{c=1}^{K}w_{c}y_{c}\log p_{c}$$

where ℒ*_ce_* denotes the cross-entropy loss, *K* is the total number of classes, *y_c_* is the ground truth indicator for class *c*, and *p_c_* is the predicted probability of class *c*. Based on this, the focal loss is further defined as:

(16) $$L_{\mathrm{focal}}={\left(1-e^{-L_{\mathrm{ce}}}\right)}^{\gamma }L_{\mathrm{ce}}$$

where ℒ*_focal_* denotes the focal loss and *γ* is the focusing parameter. The main role of this loss is to reduce the contribution of easily classified samples to the overall loss, thereby encouraging the model to place greater emphasis on hard-to-classify samples and minority class samples.

For optimization, Adam is adopted together with a learning rate schedule that combines linear warmup and cosine annealing. Let the learning rate at epoch *e* be *η_e_*, the initial learning rate be *η*_0_, the minimum learning rate be *η*_min_, the total number of training epochs be *E*, and the number of warmup epochs be *E_w_*. The learning rate is updated as:

(17) $$\eta_{e}=\left\{\begin{array}{ll} \eta_{0}\frac{e}{E_{w}}, & e\leq E_{w}\\ \eta_{\min}+\frac{\eta_{0}-\eta_{\min}}{2}\left[1+\cos\left(\pi \frac{e-E_{w}}{E-E_{w}}\right)\right], & e>E_{w} \end{array}\right.$$

By integrating automatic class weighting, weighted sampling, focal loss, and this learning rate scheduling strategy, the proposed method forms a complete optimization scheme for clinical long-tailed data.

## 4. Experiment and Analysis

### 4.1. Experimental Setup and Evaluation Metrics

**Figure 4:**
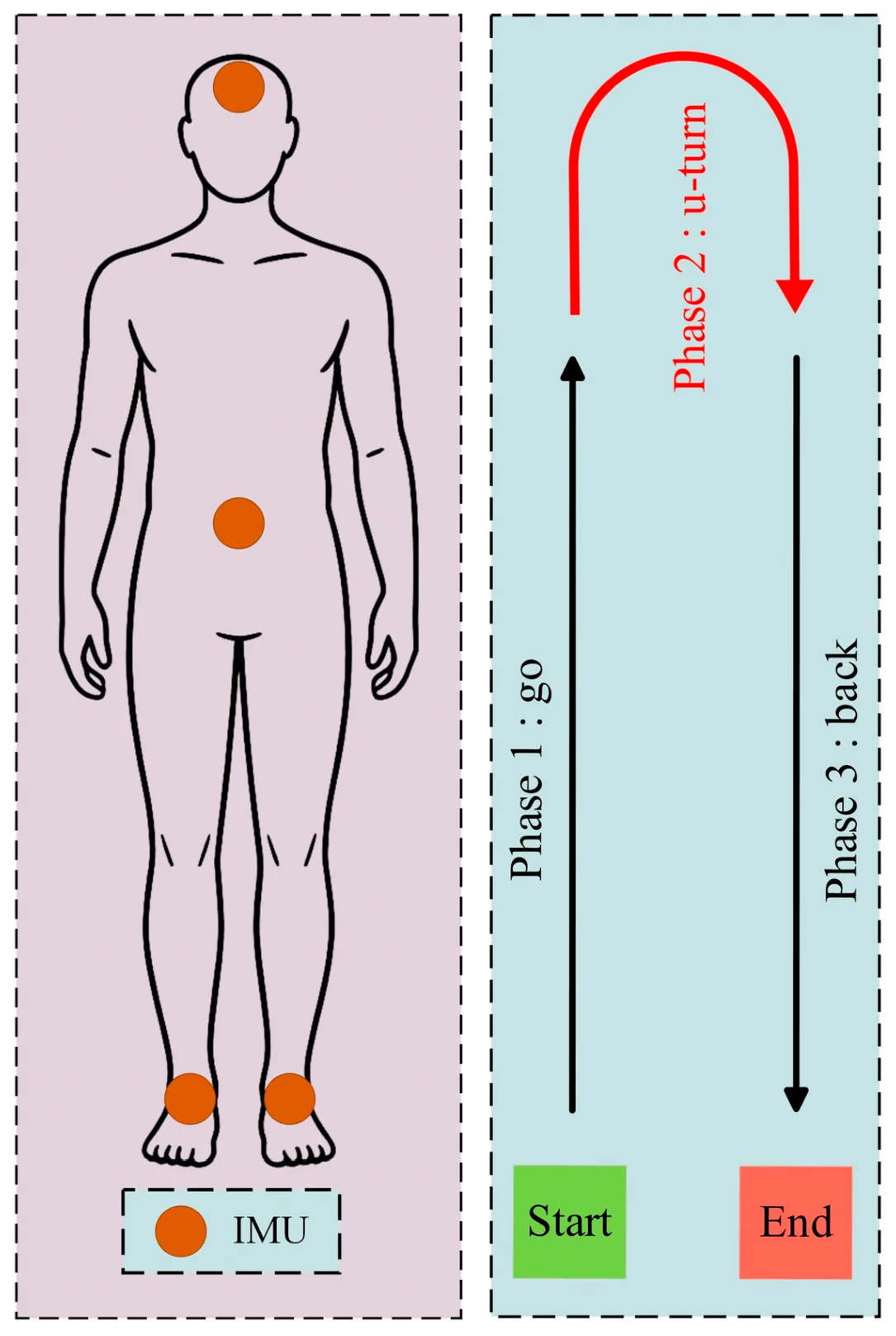
Illustration of IMU placement and gait trial protocol in the public dataset. Four IMUs were placed on the head, lower back, and the dorsal side of both feet. In each trial, the participant started from a standing position, walked forward, performed a U-turn, and then walked back to the end point.

This study conducts experimental investigations using a publicly available clinical gait dataset [20]. The dataset includes inertial gait signals collected from healthy individuals as well as subjects with various neurological and orthopedic disorders, covering eight categories: healthy controls, hip osteoarthritis, knee osteoarthritis, anterior cruciate ligament injury, stroke, Parkinson’s disease, chemotherapy-induced peripheral neuropathy, and radiation-induced leukoencephalopathy. It therefore provides a relatively rich clinical sample basis for multi-class lower-limb disease gait recognition. The experiment adopted a standardized acquisition protocol to obtain synchronized inertial gait data from multiple body locations, as shown in Figure 4. Compared with analyzing only straight walking, this protocol can further capture the dynamic adjustment characteristics during the turning phase, thereby improving the analysis of abnormal gait patterns.

All experiments in this study were conducted under a unified hardware and software environment. The experimental platform was configured with an Intel i5-14600KF processor (Intel Corporation, Santa Clara, CA, USA), 32 GB of memory, and an NVIDIA RTX 5070 graphics processing unit (NVIDIA Corporation, Santa Clara, CA, USA). The deep learning framework used was PyTorch 2.8.0, and the Python version was 3.10. Each model and ablation configuration was evaluated in five training runs using random seeds of 42, 2025, 2026, 2027, and 3407. The subject-independent split was held fixed across all runs, with the same subject and sample assignments as reported in Table 1 and Table 2. Classification metrics in Table 3, Table 4, Table 5, Table 6, Table 7 and Table 8 are reported as mean ± population standard deviation across the five runs, with the standard deviation calculated using a denominator of five.

The 260 subjects were randomly assigned to the training, validation, and test sets in proportions of 70%, 15%, and 15%, respectively. All gait trials from the same subject were assigned to the same set, ensuring a subject-independent split. The training, validation, and test sets contained 182, 39, and 39 subjects and 899, 222, and 235 input samples, respectively. The class-wise subject and sample counts are presented in Table 1 and Table 2. For the input signals, uniform length processing was first performed, followed by normalization based on the statistics of the training set. During training, model parameters were updated using mini-batch iteration, with a batch size of 64, an input sequence length of 2048, an initial learning rate of 0.0003, the Adam optimizer, and 400 training epochs.

The input length of 2048 time steps provides a uniform format for mini-batch processing. At this length, 1242 of the 1356 preprocessed gait sequences (91.59%) retained all valid time steps and required only zero-padding, whereas 114 sequences (8.41%) required center cropping. The training, validation, and test sets contained 73, 38, and 3 cropped sequences and 826, 184, and 232 zero-padded sequences, respectively.

Center cropping discarded 114,137 of the 1,890,143 valid preprocessed time steps, corresponding to an overall reduction of 6.04%. The proportions of valid preprocessed time steps discarded in the training, validation, and test sets were 6.15%, 9.95%, and 1.05%, respectively. Among the 114 cropped sequences, the mean and maximum sequence-level discarded proportions were 24.20% and 77.79%, respectively. Center cropping may therefore exclude gait events or transitions outside the retained central segment, particularly in longer sequences.

To comprehensively evaluate model performance on the multiclass lower limb disease gait recognition task, Accuracy, Macro Precision, Macro Recall, and Macro F1 were adopted as the main evaluation metrics. Among them, Accuracy reflects the overall classification correctness of the model and is defined as follows:

(18) $$\mathrm{Accuracy}=\frac{\sum_{i=1}^{C}TP_{i}}{N}$$

where *C* denotes the total number of classes, *TPi* denotes the number of correctly classified samples in class *i* and *N* denotes the total number of samples in the test set.

For class *i* Precision and Recall are defined as follows:

(19) $$P_{i}=\frac{TP_{i}}{TP_{i}+FP_{i}}$$

(20) $$R_{i}=\frac{TP_{i}}{TP_{i}+FN_{i}}$$

where *FP_i_* denotes the number of samples incorrectly predicted as class *i* and *FN_i_* denotes the number of samples that truly belong to class *i* but are misclassified into other classes.

Furthermore, the F1 score for class *i* is defined as follows:

(21) $$F1_{i}=\frac{2P_{i}R_{i}}{P_{i}+R_{i}}$$

Based on these definitions, Macro-Precision, Macro-Recall, and Macro-F1 are respectively defined as follows:

(22) $$\mathrm{Macro}-\mathrm{Precision}=\frac{1}{C}\sum_{i=1}^{C}P_{i}$$

(23) $$\mathrm{Macro}-\mathrm{Recall}=\frac{1}{C}\sum_{i=1}^{C}R_{i}$$

(24) $$\mathrm{Macro}-F1=\frac{1}{C}\sum_{i=1}^{C}F1_{i}$$

Among these metrics, Accuracy mainly measures the overall classification capability of the model, while Macro Precision, Macro Recall, and Macro F1 assign equal importance to all classes and therefore better reflect the balance of recognition performance across categories, especially for minority classes and classes that are more difficult to distinguish.

### 4.2. Comparison with Baseline Models

Table 3 presents the comparative results of the proposed method and the baseline models on the multi-class lower-limb disease gait recognition task. Overall, the proposed method achieves the highest mean values across all four evaluation metrics in Table 3. Its advantage is reflected not only in the improvement of overall classification accuracy, but also in a better balance of recognition performance across different categories. In particular, the gains in macro-recall and macro-F1 further indicate that the proposed method is more effective in mitigating the interference caused by class imbalance and inter-class similarity, rather than simply obtaining higher accuracy through sufficient fitting of majority-class samples.

The performance differences among the baseline models also show that convolution-based temporal models, such as ResNet1D and TCN, generally outperform CNN-LSTM and Transformer-Encoder. This suggests that, for IMU-based gait signals, local temporal continuity and dynamic evolution patterns at different time scales provide important discriminative cues for disease classification. Building upon temporal modeling, the proposed method further strengthens the exploitation of structural relationships among multiple sensors, thereby enabling more effective characterization of abnormal coordination patterns, rhythm shifts, and compensatory coupling features across different acquisition sites, including the head, lower back, and both feet. Meanwhile, the proposed model still maintains relatively low parameter count and computational cost, indicating that its performance improvement mainly arises from more effective feature organization and a more reasonable structural design, rather than from a simple expansion of model size.

**Figure 5:**
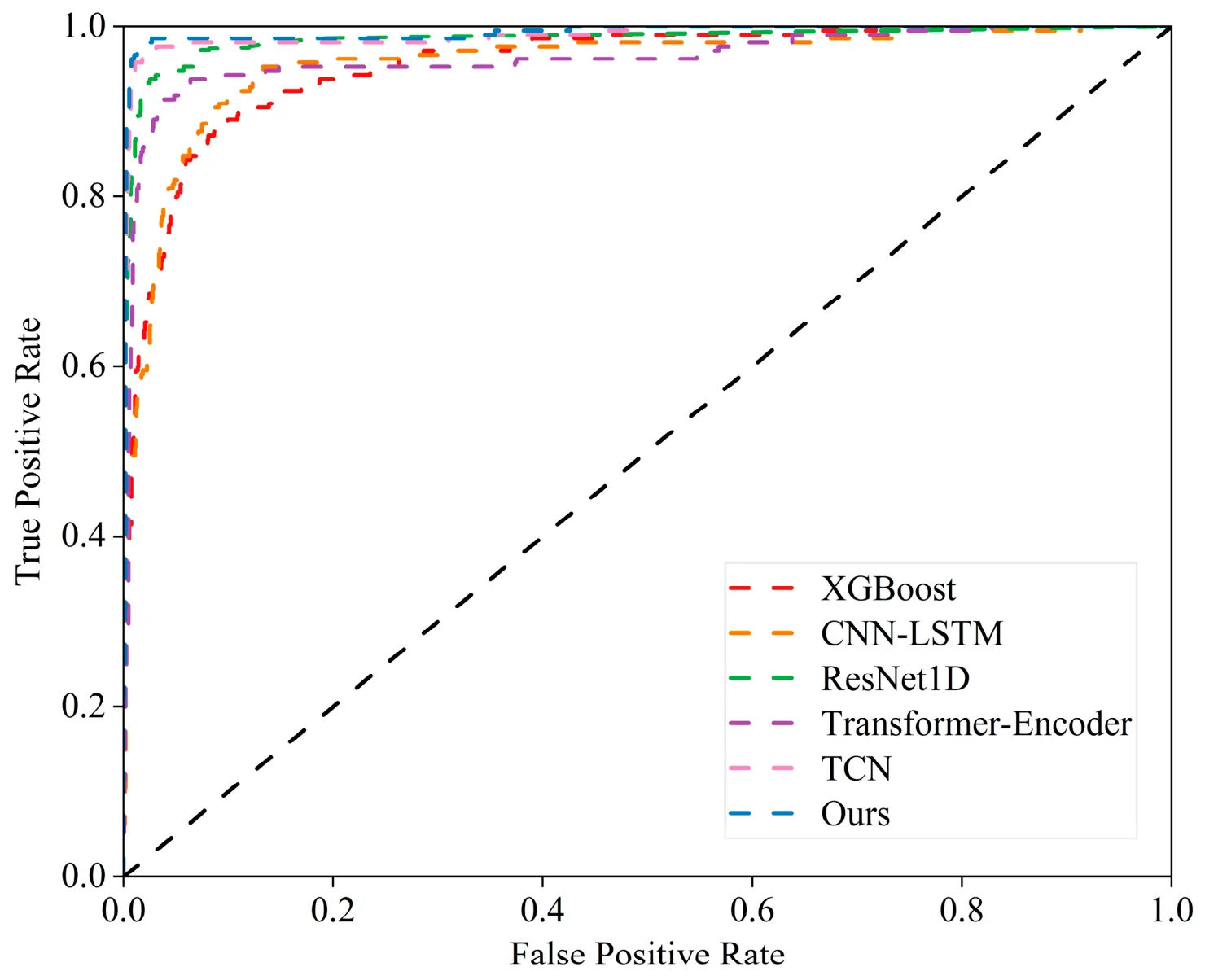
Macro-averaged one-vs-rest ROC curves of TSDGNet and baseline models for eight-class pathological gait classification.

For the eight-class classification task, Figure 5 presents macro-averaged one-vs-rest (OvR) ROC curves. For each model, the class-specific true positive rates were linearly interpolated onto a common false positive rate grid and averaged with equal weights across all eight classes. As shown by the ROC curves in Figure 5, the curve of the proposed method lies closest to the upper-left corner overall, while maintaining a high true positive rate and a low false positive rate over a wide threshold range. This demonstrates that the model possesses strong stability and robustness under different decision thresholds. In contrast, the ROC curves of the other baseline models are generally shifted slightly toward the lower-right region, suggesting that their ability to characterize complex class boundaries is relatively limited. This observation is consistent with the quantitative results in Table 3 and further confirms the overall discriminative advantage of the proposed method in the multi-class lower-limb disease gait recognition task.

**Figure 6:**
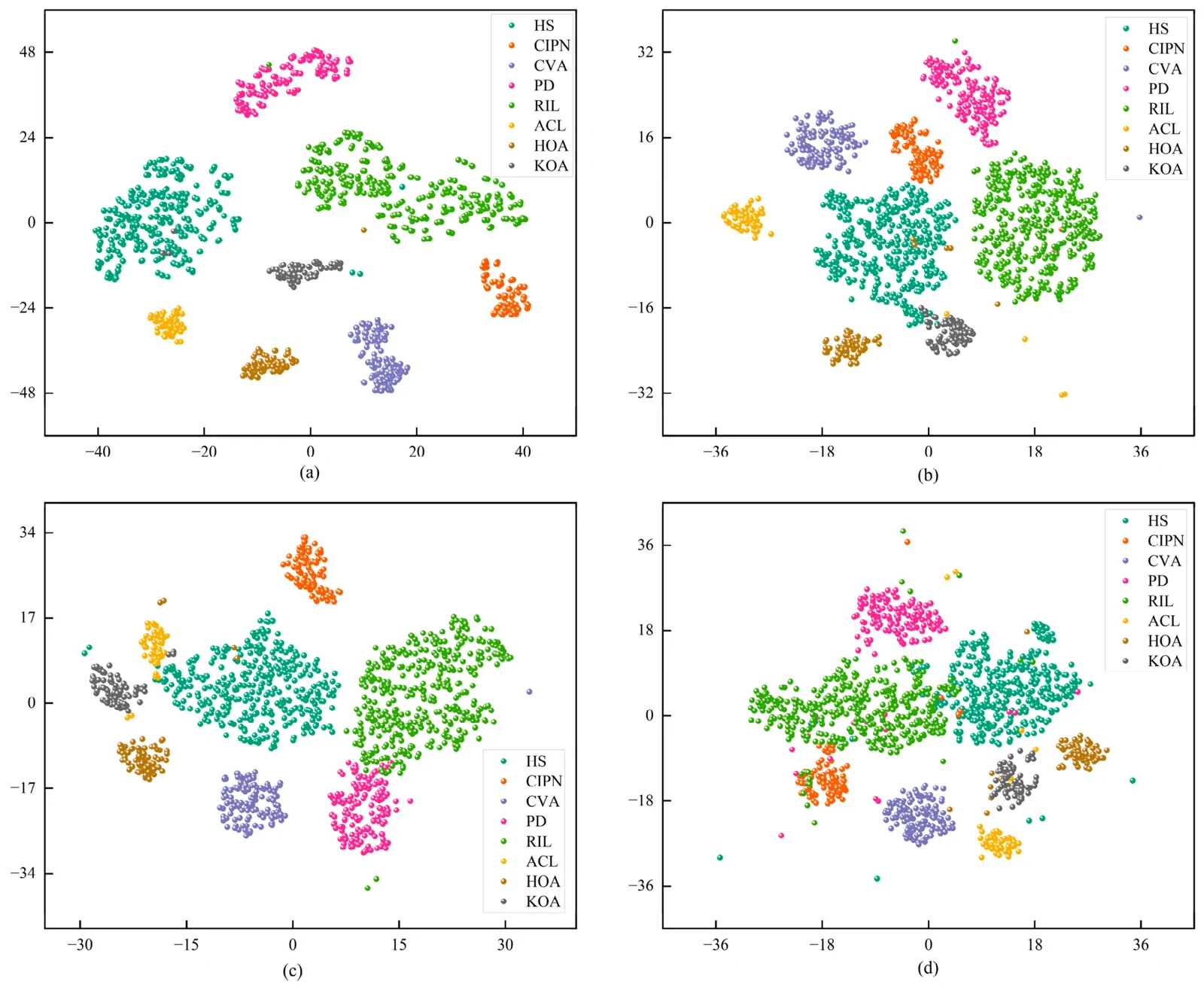
t-SNE visualization of feature representations learned by TSDGNet and baseline models for the multi-class lower-limb disease gait recognition task: (**a**) TSDGNet, (**b**) TCN, (**c**) ResNet1D, and (**d**) Transformer-Encoder.

Figure 6 illustrates the t-SNE projections of the features learned by different models in a two-dimensional space. It can be observed that the features produced by the proposed method exhibit a clearer category-wise organization. Most classes form relatively compact and independent clusters in the embedding space, with more distinct inter-cluster margins and fewer outliers. By comparison, although TCN and ResNet1D can also separate some categories to a certain extent, several classes still remain close to one another in local regions, with partial overlap and less well-defined cluster boundaries. The feature distribution learned by Transformer-Encoder is even more scattered, with more pronounced inter-class mixing.

Further observation reveals that, under the proposed method, certain categories not only exhibit greater compactness within classes but also demonstrate more stable spatial distributions across different classes, indicating that the model is capable of mapping raw gait signals into a more discriminative feature space. In contrast, the remaining baseline models still suffer from elongated distributions within classes, insufficient margins between classes, or localized class overlap. This distributional discrepancy is consistent with the quantitative trends reported in Table 3, suggesting that the proposed method effectively learns more discriminative feature representations, thereby providing a more stable and robust representational foundation for recognition of lower limb disorders across multiple categories.

### 4.3. Comparison with Advanced Models

**Figure 7:**
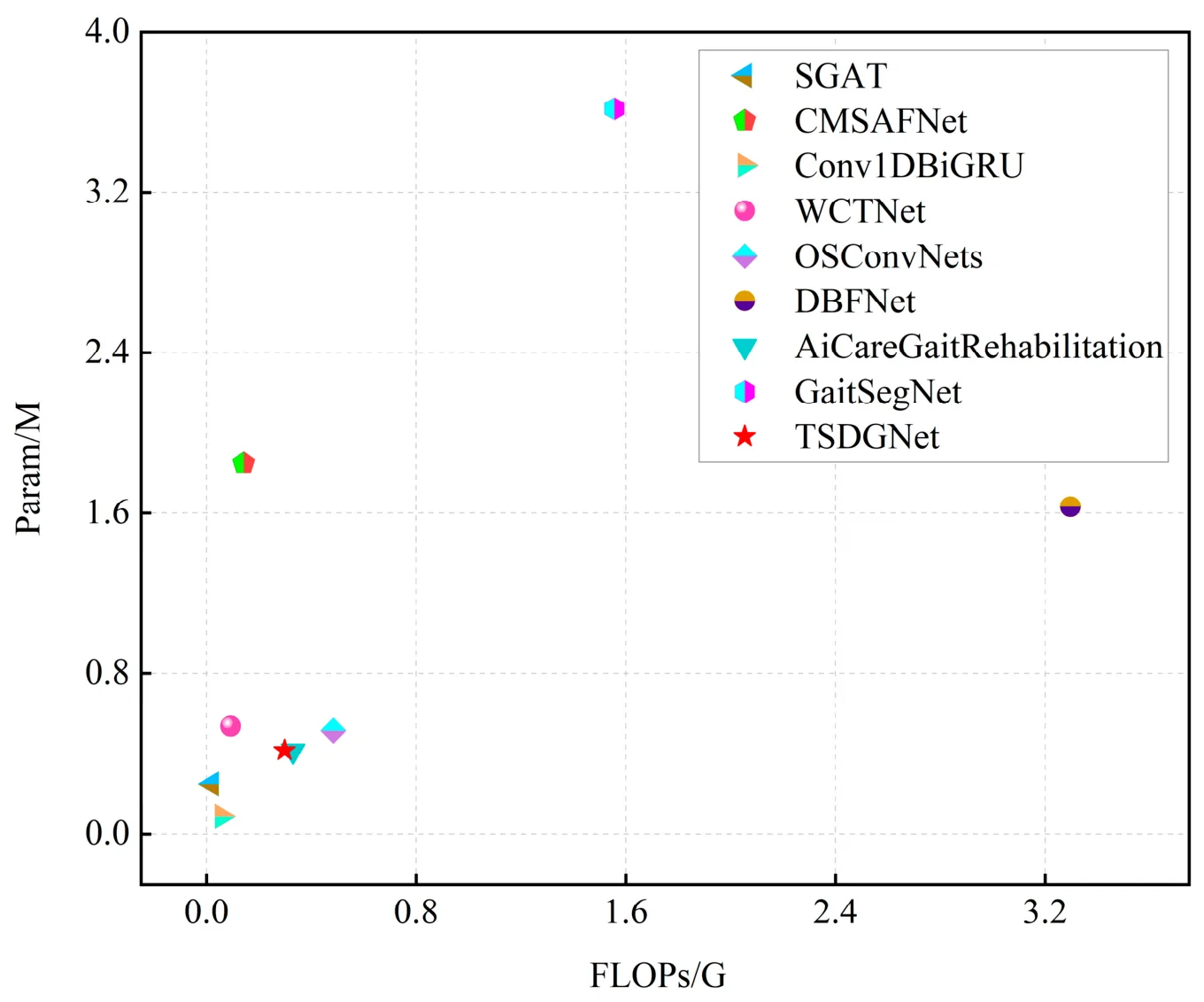
Distribution of different models in terms of parameter scale and computational complexity. The horizontal axis denotes FLOPs (G), and the vertical axis denotes Param (M).

A comparison with representative advanced models was conducted to assess the classification performance of TSDGNet under the same subject-independent evaluation protocol. Table 4 summarizes the mean performance and population standard deviation across five training runs, while Figure 7 presents the parameter counts and computational costs of the evaluated models. TSDGNet achieves the highest mean values across all four classification metrics, with Accuracy, Macro-Precision, Macro-Recall, and Macro-F1 of 97.2 ± 0.58%, 96.2 ± 1.05%, 97.1 ± 1.24%, and 95.3 ± 0.60%, respectively. These results show favorable average performance across both overall accuracy and the macro-averaged evaluation metrics.

Among the competing models, GaitSegNet achieves the highest mean Macro-F1 at 94.1 ± 0.92%, compared with 95.3 ± 0.60% for TSDGNet, giving a mean difference of 1.2 percentage points. TSDGNet also exceeds GaitSegNet in mean Accuracy, Macro-Precision, and Macro-Recall by 1.6, 1.5, and 1.8 percentage points, respectively. The improvements in the macro-averaged metrics are relevant to this imbalanced classification task because each category contributes equally to these measures, allowing performance across the eight categories to be assessed alongside overall accuracy. The comparisons describe average performance across five training seeds on the fixed subject-independent split, and the accompanying standard deviations characterize the variability observed across these runs.

In addition to recognition performance, model efficiency was also considered. As shown in Table 4, TSDGNet requires only 0.419 M parameters and 0.2981 G FLOPs, remaining at a relatively low complexity level. The same trend can be observed more intuitively in Figure 7, where TSDGNet is located in a relatively low complexity region while still corresponding to superior performance in Table 4. By contrast, models such as GaitSegNet and DBFNet are distributed in regions with noticeably higher parameter burden and computational cost, whereas lightweight methods such as SGAT and Conv1DBiGRU, although computationally cheaper, still show an evident performance gap. This suggests that the improvement of TSDGNet does not mainly rely on enlarging model size, but rather on more effective modeling of pathology-related temporal dynamics and structural relationships between body segments.

Overall, the joint evidence from Table 4 and Figure 7 shows that TSDGNet achieves a more favorable tradeoff between recognition performance and computational efficiency. Such a characteristic is particularly valuable for clinical auxiliary recognition scenarios, where both classification reliability and deployment cost need to be considered simultaneously.

### 4.4. Effect of Temporal Receptive Field Configurations

To investigate the effect of different temporal receptive field configurations in the temporal selective kernel branch, three model variants were constructed by modifying only the kernel settings in the temporal branch, while keeping the dual-graph learning module, optimization strategy, and all other experimental settings unchanged. Specifically, TSDGNet-a, TSDGNet-b, and TSDGNet-c correspond to kernel configurations of (1,3,5), (3,5,7), and (5,7,9), respectively, whereas the full TSDGNet adopts the configuration of (3,5,9). Under this controlled setting, the influence of temporal receptive field configurations on multi-class lower-limb disease gait recognition can be examined more directly.

As shown in Table 5, TSDGNet achieves the highest mean values across all four evaluation metrics among the evaluated kernel configurations. TSDGNet-a places greater emphasis on modeling short-term local variations, which helps capture more subtle transient changes. However, its receptive field is relatively limited, making it less effective in exploiting longer-range rhythmic variations and the dynamic associations across different gait phases. In contrast, TSDGNet-c expands the temporal coverage and strengthens the modeling of overall dynamic patterns, but this tendency also weakens the model’s sensitivity to fine-grained local abnormalities, thereby reducing its ability to distinguish categories with subtle pathological differences. Compared with these two variants, TSDGNet-b provides a more balanced temporal modeling scheme and therefore achieves better performance, suggesting that mid-scale temporal patterns already contain considerable discriminative information. Nevertheless, its performance still falls short of that of the full TSDGNet, indicating that without the support of a larger temporal receptive field, the model remains insufficient in capturing dependencies over a broader temporal range.

A further analysis with model complexity shows that the performance gain does not result from a simple increase in the number of parameters or FLOPs. Although TSDGNet-c has the highest computational cost, it does not achieve the best recognition performance. By contrast, TSDGNet is not the most complex variant, yet it delivers the best results. This suggests that the key factor in this experiment is not merely enlarging the model scale, but whether different temporal scales can complement each other effectively. Overall, the configuration adopted in TSDGNet achieves a more appropriate balance between preserving local fine-grained information and modeling overall dynamic patterns, which is critical for accurate multi-class lower-limb disease gait recognition.

### 4.5. Ablation Experiment

To examine the contributions of the main model branches and their individual design choices, we performed branch-level ablations in Table 6 and component-level ablations in Table 7 and Table 8 under the same experimental protocol. Specifically, K denotes the full model, L denotes the model without the graph branch, M denotes the model without the temporal branch, and N denotes the model without the class imbalance optimization. This design makes it possible to separately examine the roles of temporal dynamic modeling, structural relationship learning, and imbalance-aware optimization within the overall framework, and to further analyze how model performance degrades when different components are removed.

As shown in Table 6, the full model K achieves the highest mean values across all four evaluation metrics. In particular, K still shows a clear performance advantage over N despite their almost identical computational complexity, indicating that the superiority of the full model does not come from additional computational cost, but rather from the joint improvement in the way discriminative information is organized and how the model is optimized. From the performance changes across the ablated settings, M exhibits the most substantial decline, suggesting that the temporal branch provides the most critical representational support for this task. The differences among pathological gaits are not mainly reflected in the deviation of a single instantaneous posture, but more often in the way rhythmic abnormalities, disordered phase transitions, and local abnormal fluctuations evolve over time. Therefore, without temporal modeling, the model struggles to capture the key discriminative information embedded in the dynamic evolution of gait. By contrast, although the degradation of L and N is less severe than that of M, the implications of these two variants are different. L indicates that relying only on temporal patterns is still insufficient to fully characterize the coordinated abnormalities across different body parts. N, on the other hand, shows that even when the main representation ability remains largely unchanged, removing optimization constraints tailored to the clinical data distribution still causes the model to develop a bias toward easy-to-classify and dominant classes during training, thereby weakening the overall balance of recognition performance.

**Figure 8:**
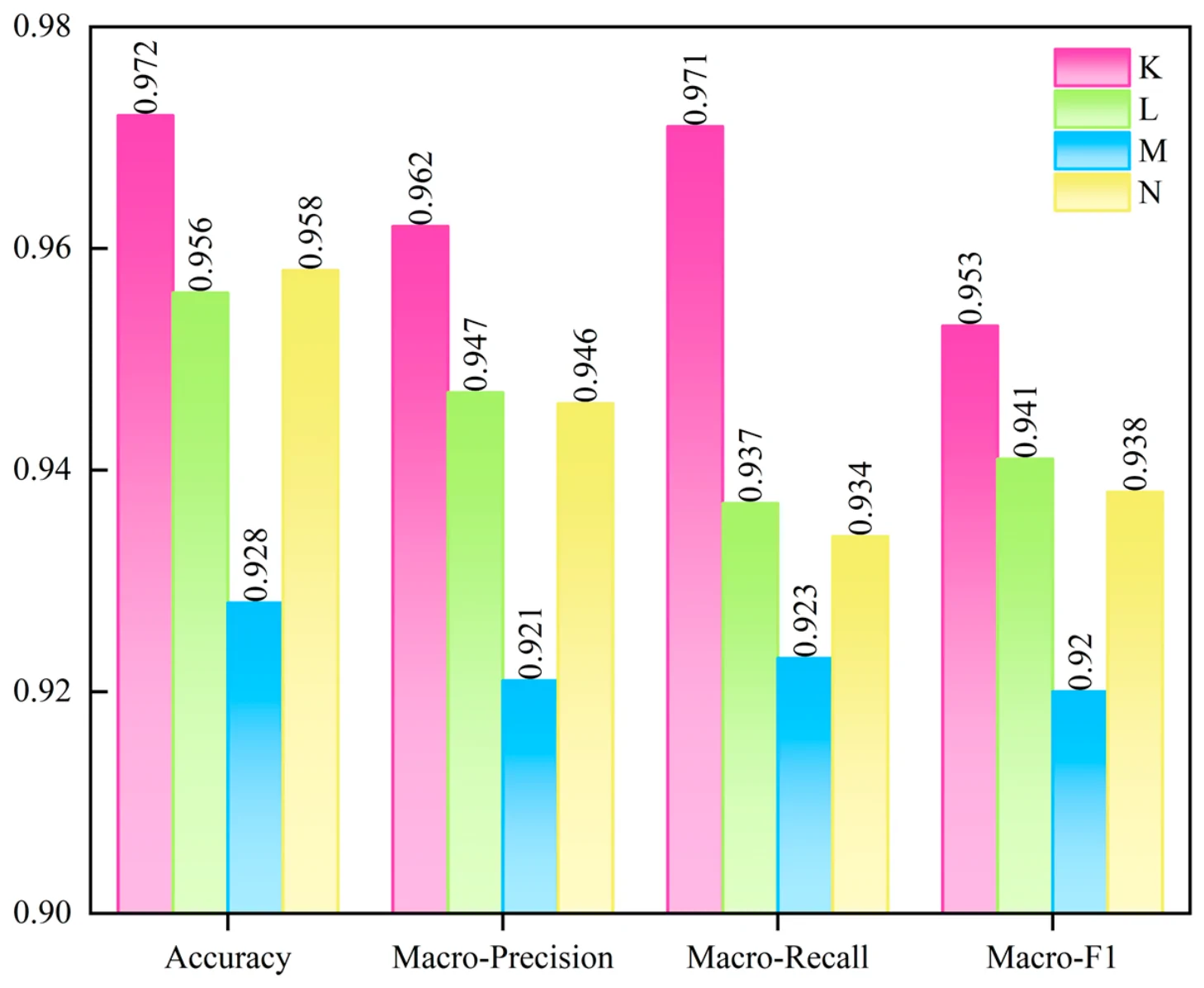
Quantitative comparison of different ablation settings on the multi-class lower-limb disease gait recognition task. K denotes the full model, L denotes the variant without the graph branch, M denotes the variant without the temporal branch, and N denotes the variant without imbalance-aware optimization.

As shown in Figure 8, the bar chart provides a more intuitive illustration of the performance trends of different ablation settings across the evaluation metrics. K remains optimal in accuracy, macro precision, macro recall, and macro F1, indicating that the complete model not only improves overall classification performance but also enhances the balance of recognition across different classes. All four metrics of M decrease simultaneously, and the downward trend is relatively consistent, suggesting that after removing the temporal branch, the damage affects the model’s fundamental discriminative ability for the task as a whole rather than causing only local degradation in a few categories. In contrast, the degradations of L and N are more directional. The change in L is mainly reflected in a weakened ability to distinguish complex inter-class relationships, which indicates that the graph branch does not replace temporal modeling, but rather further integrates coordinated cross-body variations on top of dynamic information. Compared with K, N shows lower mean scores across all four evaluation metrics, with Macro-Recall showing the largest decrease of 3.7 percentage points.

**Figure 9:**
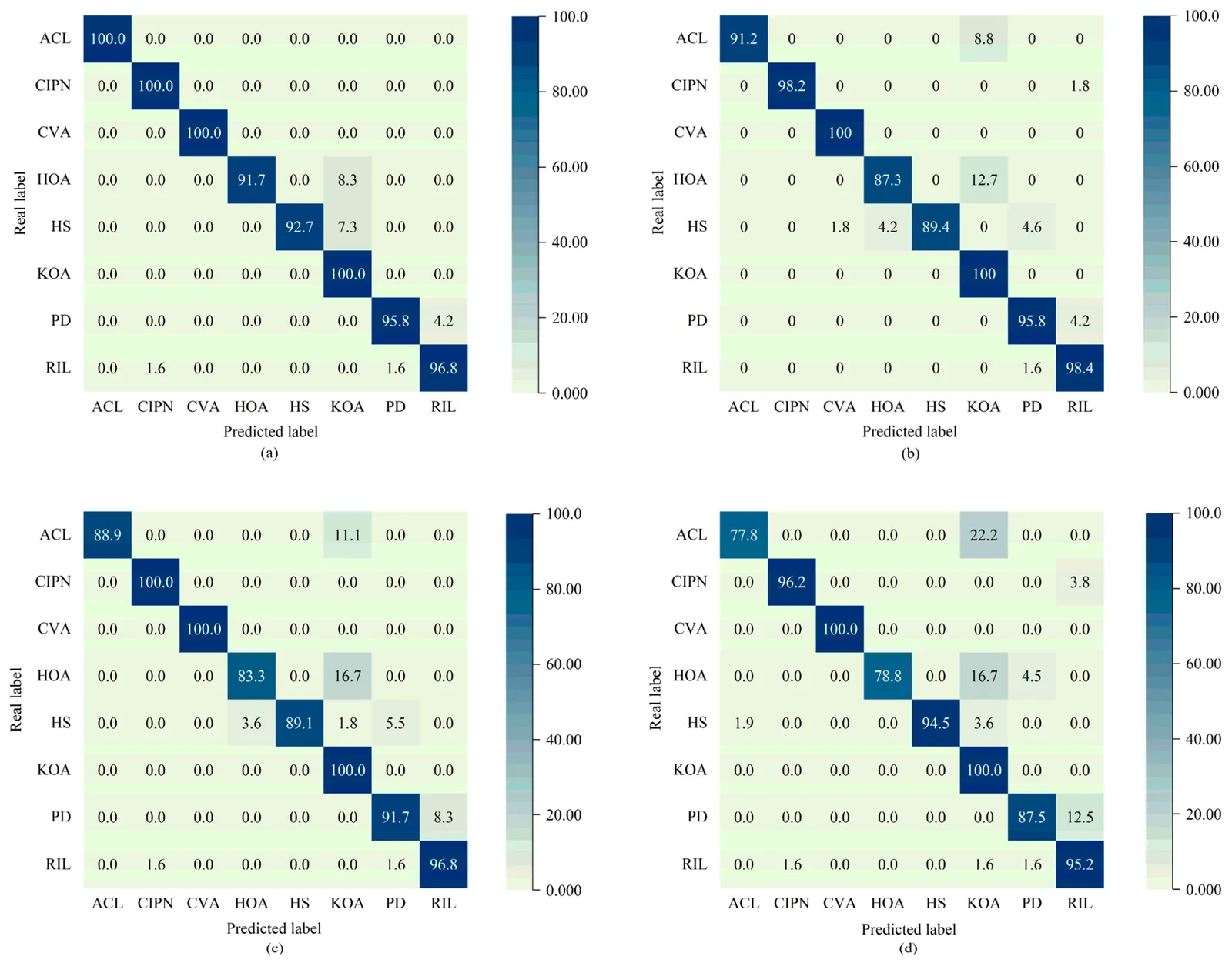
Confusion matrices of different ablation settings for the multi-class lower-limb disease gait recognition task: (**a**) full model, (**b**) variant without the graph branch, (**c**) variant without imbalance-aware optimization, and (**d**) variant without the temporal branch.

As shown in Figure 9, the four confusion matrices further reveal the distribution patterns of misclassification after different modules are removed. The predictions of K are mostly concentrated along the main diagonal, with only slight confusion between a few similar categories, indicating that the full model can already distinguish multiple pathological gait types in a relatively stable manner. After removing the graph branch, misclassifications are more likely to concentrate among several categories with similar compensatory patterns, especially with more evident confusion from HOA to KOA and from PD to RIL. This suggests that when relying only on temporal features, the model can detect the presence of abnormalities, but finds it difficult to further identify how these abnormalities are expressed through coordination imbalance among different body parts, whereas the graph branch provides precisely this layer of structural information. After removing the temporal branch, the range of confusion expands further, and misclassifications are no longer limited to a few categories but spread across multiple similar pathologies, indicating that structural relationships alone are insufficient for fine-grained discrimination. The reason is that structural relationships can reflect whether different body parts exhibit shared abnormalities, but cannot adequately describe how these abnormalities persist, shift, and gradually accumulate throughout the gait cycle. After removing the class imbalance optimization, the misclassification of several difficult categories increases again, and the error distribution becomes more scattered, indicating that the main role of this strategy is not to improve the separability of easy classes, but to stabilize the decision boundaries of difficult classes.

**Figure 10:**
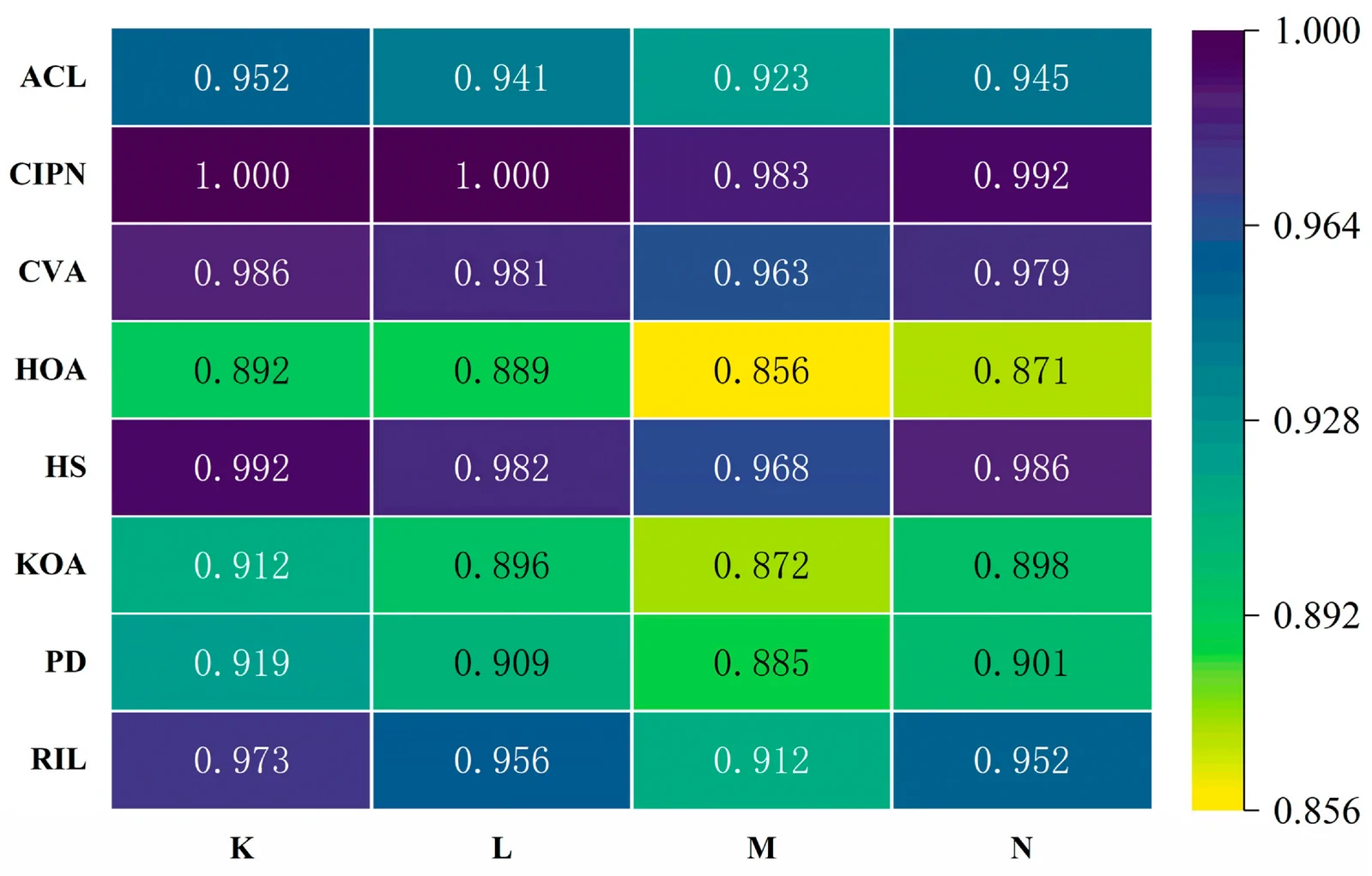
Heatmap of class-wise F1-scores for different ablation settings on the multi-class lower-limb disease gait recognition task, showing the class-dependent performance changes after removing different components.

As shown in Figure 10, the class-level F1 heatmap further confirms the above observations at the category level. K maintains more stable F1 scores across most classes, indicating that the advantage of the full model does not rely on isolated improvements in only a few categories, but is reflected in a more balanced performance gain across the full set of classes. Compared with K, the decline in L is mainly concentrated in several categories that are more difficult to distinguish, suggesting that the graph branch contributes not only to overall performance improvement but also to a stronger ability to resolve complex inter-class relationships. N also shows more pronounced declines in difficult categories, indicating that imbalance optimization does not significantly change the upper limit of performance for easy classes, but can alleviate the adverse effect of class distribution bias on minority and disadvantaged classes during training. By contrast, the degradation of M is much more pervasive. Its effect is not restricted to a few individual categories, but extends across multiple classes, further demonstrating that once the temporal branch is removed, what is lost is not merely the recognition ability for certain local categories, but the overall ability to differentiate complex pathological dynamics.

Table 7 extends the temporal kernel comparisons in Section 4.4 by evaluating single-scale controls and individual graph components. Each single-scale variant retains one convolutional branch in each temporal module, using a kernel size of 3, 5, or 9 while preserving the output feature dimensions. The single-graph variants retain only the anatomical prior graph or the symbiotic prior graph. For the variant without context-guided gating, the residual increments from the two graph branches are averaged with equal weights. For the variant without graph attention, attention-based propagation is replaced by normalized neighborhood aggregation over the same fixed adjacency structures, while linear feature mapping and subsequent residual updating are retained. The imbalance optimization strategy and remaining training settings are held fixed.

As shown in Table 7, replacing the multiscale temporal module with a single-scale branch reduces mean Accuracy by 2.0, 2.7, and 2.5 percentage points for kernel sizes of 3, 5, and 9, respectively. The corresponding reductions in mean Macro-F1 are 1.3, 1.8, and 1.1 percentage points. Across these evaluated temporal configurations, the multiscale model achieves higher mean classification scores than each single-scale counterpart, supporting the use of multiple temporal scales within the proposed architecture.

The anatomical-only and symbiotic-only variants obtain mean Macro-F1 scores of 94.2% and 93.6%, respectively, compared with 95.3% for the full model. Removing context-guided gating reduces mean Accuracy to 95.3% and mean Macro-F1 to 94.3%. Replacing graph attention with fixed normalized neighborhood aggregation gives mean Accuracy of 96.4% and mean Macro-F1 of 95.0%. These comparisons show the performance changes associated with restricting the graph representation to one prior structure, replacing adaptive residual fusion with equal weighting, and replacing learned attention with fixed aggregation.

Table 8 evaluates the individual components of the imbalance optimization strategy. Removing class weighting retains focal modulation and weighted sampling. Removing weighted sampling uses uniform sampling while retaining the class-weighted focal loss. Removing focal modulation replaces the focal loss with class-weighted cross-entropy while retaining weighted sampling. The configuration without imbalance optimization uses unweighted cross-entropy and uniform sampling. The model architecture, subject partition, and remaining training settings are held fixed across these comparisons.

As shown in Table 8, the full optimization strategy achieves the highest reported mean values across all four classification metrics. Removing class weighting, weighted sampling, and focal modulation reduces mean Accuracy by 1.0, 0.9, and 1.1 percentage points, respectively. The corresponding reductions in mean Macro-Recall are 1.8, 2.3, and 1.8 percentage points, with removal of weighted sampling producing the largest decrease among the three individual ablations. Mean Macro-F1 decreases by 0.1, 0.2, and 0.3 percentage points, respectively. Removing the entire imbalance optimization strategy produces larger reductions of 3.7 percentage points in Macro-Recall and 1.5 percentage points in Macro-F1. The individual removal comparisons quantify the contribution of each technique when the remaining optimization components are retained.

Taken together, the temporal branch, graph branch, and class imbalance optimization operate on three different levels, namely dynamic representation, structural discrimination, and training bias control. They are not parallel modules that can simply substitute for one another, but instead play sequential and complementary roles within the same recognition chain. Within the evaluated ablation settings, the full configuration achieves the highest mean values across all four classification metrics in Table 6, Table 7 and Table 8.

The fixed test set contains two KOA trials from one subject and five ACL trials from one subject. Within a single run, each additional misclassified trial reduces class-specific recall by 50 percentage points for KOA and 20 percentage points for ACL. All five training runs use the same test subjects and trials, and the reported standard deviations quantify variation across training seeds under this fixed subject-independent split.

## 5. Conclusions

A TSDGNet-based approach is developed for multi-class lower limb disease gait recognition to address substantial inter-subject variability, similar pathological manifestations, and class imbalance. The proposed method combines multiscale temporal feature extraction with sequence-level cross-sensor feature refinement using fixed graph priors and context-guided residual updates. In addition, a class-balancing optimization strategy is introduced to improve the recognition of minority classes and easily confused categories. The experimental results suggest the potential of the proposed approach for multiclass pathological gait classification on the evaluated public dataset. However, evaluation was limited to a single dataset, and generalizability to different sensor placements, acquisition protocols, and patient populations remains unverified. External validation and prospective clinical evaluation are needed to assess broader generalizability and clinical applicability.

## Figures and Tables

**Table 1:** Class-wise subject counts in the training, validation, and test sets.

Class	Training	Validation	Test	Total
HS	55	6	12	73
HOA	9	4	2	15
KOA	15	2	1	18
ACL	8	2	1	11
CVA	35	7	7	49
PD	17	2	5	24
CIPN	14	3	2	19
RIL	29	13	9	51
Total	182	39	39	260

Note: Subjects were randomly assigned to the training, validation, and test sets in proportions of 70%, 15%, and 15%, respectively, with no subject shared across sets. HS, healthy subjects; HOA, hip osteoarthritis; KOA, knee osteoarthritis; ACL, anterior cruciate ligament injury; CVA, cerebrovascular accident; PD, Parkinson’s disease; CIPN, chemotherapy-induced peripheral neuropathy; RIL, radiation-induced leukoencephalopathy.

**Table 2:** Class-wise sample counts in the training, validation, and test sets.

Class	Training	Validation	Test	Total
HS	251	29	80	360
HOA	40	20	14	74
KOA	63	13	2	78
ACL	43	12	5	60
CVA	84	16	28	128
PD	115	10	35	160
CIPN	73	15	10	98
RIL	230	107	61	398
Total	899	222	235	1356

Note: Each gait trial corresponds to one model input sample standardized to 2048 time steps. Longer sequences were center-cropped, whereas shorter sequences were zero-padded at the end. HS, healthy subjects; HOA, hip osteoarthritis; KOA, knee osteoarthritis; ACL, anterior cruciate ligament injury; CVA, cerebrovascular accident; PD, Parkinson’s disease; CIPN, chemotherapy-induced peripheral neuropathy; RIL, radiation-induced leukoencephalopathy.

**Table 3:** Performance comparison of TSDGNet and baseline models on the multi-class lower-limb disease gait recognition task.

Method	Acc.	Macro-P	Macro-R	Macro-F1	Params	FLOPs
XGBoost	76.3 ± 1.02	74.6 ± 0.99	73.9 ± 0.73	74.1 ± 1.24	-	-
ResNet1D	91.6 ± 0.68	92.3 ± 1.15	90.8 ± 1.07	89.9 ± 0.88	1.902	1.3025
TCN	93.3 ± 1.36	93.2 ± 0.54	91.4 ± 1.21	90.2 ± 1.25	1.636	6.4069
CNN-LSTM	81.4 ± 0.64	83.1 ± 1.02	78.9 ± 0.78	79.1 ± 1.06	0.829	0.0557
Transformer-Encoder	86.7 ± 1.27	83.9 ± 1.07	86.2 ± 0.53	84.5 ± 1.21	0.601	0.0556
Ours	97.2 ± 0.58	96.2 ± 1.05	97.1 ± 1.24	95.3 ± 0.60	0.419	0.2981

Note: Acc., accuracy; Macro-P, macro-precision; Macro-R, macro-recall; Macro-F1, macro-F1 score. Classification metrics (%) are reported as mean ± population standard deviation across five training runs on the same fixed subject-independent split. Params and FLOPs are reported in M and G, respectively.

**Table 4:** Performance comparison of TSDGNet and representative advanced models on the multi-class lower-limb disease gait recognition task.

Models	Acc.	Macro-P.	Macro-R.	Macro-F1	Params	FLOPs
SGAT [24]	80.9 ± 0.91	79.5 ± 1.31	80.4 ± 0.96	78.1 ± 0.99	0.251	0.0195
CMSAFNet [12]	81.7 ± 1.37	81.2 ± 0.58	80.3 ± 0.59	79.6 ± 1.31	1.849	0.1423
Conv1DBiGRU [36]	84.5 ± 1.22	83.4 ± 1.29	84.1 ± 1.27	82.6 ± 1.37	0.086	0.0577
WCTNet [37]	86.8 ± 1.04	84.6 ± 0.88	85.9 ± 0.62	85.1 ± 1.24	0.539	0.0916
OSConvNets [38]	90.4 ± 1.33	89.2 ± 1.25	90.2 ± 1.02	88.7 ± 0.98	0.518	0.4837
DBFNet [39]	92.1 ± 0.58	90.9 ± 0.83	91.5 ± 1.01	90.1 ± 1.01	1.631	3.2972
AiCareGaitRehabilitation [40]	95.2 ± 0.91	94.1 ± 0.58	95.7 ± 0.82	93.5 ± 0.58	0.416	0.3294
GaitSegNet [10]	95.6 ± 1.31	94.7 ± 1.06	95.3 ± 0.56	94.1 ± 0.92	3.618	1.5562
TSDGNet	97.2 ± 0.58	96.2 ± 1.05	97.1 ± 1.24	95.3 ± 0.60	0.419	0.2981

Note: Acc., accuracy; Macro-P, macro-precision; Macro-R, macro-recall; Macro-F1, macro-F1 score. Classification metrics (%) are reported as mean ± population standard deviation across five training runs on the same fixed subject-independent split. Params and FLOPs are reported in M and G, respectively. DBFNet denotes Dual-Branch Fusion Network.

**Table 5:** Effect of different kernel-size combinations in the temporal selective kernel branch.

Kernels	Acc.	Macro-P	Macro-R	Macro-F1	Params	FLOPs
TSDGNet-a	92.5 ± 1.10	90.9 ± 1.03	92.3 ± 0.59	91.6 ± 0.94	0.334	0.1849
TSDGNet-b	95.7 ± 1.29	93.4 ± 1.12	93.7 ± 0.75	93.5 ± 1.19	0.398	0.2698
TSDGNet-c	93.3 ± 1.06	92.5 ± 0.76	91.7 ± 1.27	92.1 ± 1.12	0.462	0.3547
TSDGNet	97.2 ± 0.58	96.2 ± 1.05	97.1 ± 1.24	95.3 ± 0.60	0.419	0.2981

Note: Acc., accuracy; Macro-P, macro-precision; Macro-R, macro-recall; Macro-F1, macro-F1 score. Classification metrics (%) are reported as mean ± population standard deviation across five training runs on the same fixed subject-independent split. Params and FLOPs are reported in M and G, respectively.

**Table 6:** Performance and complexity comparison under different ablation settings.

Models	Acc.	Macro-P.	Macro-R.	Macro-F1	Params	FLOPs
K	97.2 ± 0.58	96.2 ± 1.05	97.1 ± 1.24	95.3 ± 0.60	0.419	0.2981
L	95.6 ± 0.69	94.7 ± 0.92	93.7 ± 1.33	94.1 ± 0.63	0.316	0.2991
M	92.8 ± 1.32	92.1 ± 1.20	92.3 ± 1.08	92.0 ± 1.32	0.354	0.2996
N	95.8 ± 1.30	94.6 ± 1.23	93.4 ± 1.06	93.8 ± 1.10	0.419	0.2997

Note: Acc., accuracy; Macro-P., macro-precision; Macro-R., macro-recall; Macro-F1, macro-F1 score. Classification metrics (%) are reported as mean ± population standard deviation across five training runs on the same fixed subject-independent split. Params and FLOPs are reported in M and G, respectively. K, L, M, and N in the figures correspond to Full model, w/o Graph branch, w/o Temporal branch, and w/o Imbalance optimization, respectively.

**Table 7:** Component ablation of the temporal and dual-graph modules.

Variant	Acc.	Macro-P	Macro-R	Macro-F1	Params (M)	FLOPs (G)
Full TSDGNet	97.2 ± 0.58	96.2 ± 1.05	97.1 ± 1.24	95.3 ± 0.60	0.419	0.2981
Single-scale temporal branch (k = 3)	95.2 ± 0.83	93.0 ± 1.55	95.8 ± 1.18	94.0 ± 0.87	0.315	0.2245
Single-scale temporal branch (k = 5)	94.5 ± 1.01	92.2 ± 1.60	95.7 ± 1.13	93.5 ± 1.23	0.373	0.2350
Single-scale temporal branch (k = 9)	94.7 ± 1.44	94.2 ± 1.65	94.6 ± 1.77	94.2 ± 1.39	0.388	0.2560
Anatomical prior graph only	94.9 ± 1.01	94.9 ± 0.47	94.2 ± 1.58	94.2 ± 1.20	0.368	0.2534
Symbiotic prior graph only	94.1 ± 0.63	93.4 ± 0.86	94.2 ± 0.61	93.6 ± 0.70	0.368	0.2534
Without context-guided gating	95.3 ± 1.47	93.5 ± 1.56	95.6 ± 0.95	94.3 ± 1.08	0.399	0.2832
Without graph attention	96.4 ± 0.43	94.5 ± 1.24	96.1 ± 1.48	95.0 ± 1.30	0.384	0.2683

Note: Acc., accuracy; Macro-P, macro-precision; Macro-R, macro-recall; Macro-F1, macro-F1 score. Metrics (%) are mean ± population standard deviation, with five as the variance denominator. Params and FLOPs are expressed in M and G, respectively.

**Table 8:** Component ablation of the class imbalance optimization strategy.

Variant	Acc.	Macro-P	Macro-R	Macro-F1
Full optimization strategy	97.2 ± 0.58	96.2 ± 1.05	97.1 ± 1.24	95.3 ± 0.60
Without class weighting	96.2 ± 0.38	95.6 ± 1.39	95.3 ± 1.29	95.2 ± 1.40
Without weighted sampling	96.3 ± 0.43	96.0 ± 1.91	94.8 ± 1.48	95.1 ± 0.97
Without focal loss	96.1 ± 0.32	95.3 ± 1.44	95.3 ± 1.51	95.0 ± 1.35
Without imbalance optimization	95.8 ± 1.30	94.6 ± 1.23	93.4 ± 1.06	93.8 ± 1.10

Note: Acc., accuracy; Macro-P, macro-precision; Macro-R, macro-recall; Macro-F1, macro-F1 score. Metrics (%) are mean ± population standard deviation, with five as the variance denominator. Class weighting denotes loss weights. Removing focal loss retains class-weighted cross-entropy and weighted sampling; removing imbalance optimization uses unweighted cross-entropy and uniform sampling.

## Data Availability

The data analyzed in this study are publicly available on Figshare at https://doi.org/10.6084/m9.figshare.28806086 and are described in Voisard et al. [20].
